# Intelligent Academic Specialties Selection in Higher Education for Ukrainian Entrants: A Recommendation System

**DOI:** 10.3390/jintelligence10020032

**Published:** 2022-05-26

**Authors:** Solomiia Fedushko, Taras Ustyianovych, Yuriy Syerov

**Affiliations:** Social Communication and Information Activity Department, Lviv Polytechnic National University, 79000 Lviv, Ukraine; taras.ustyianovych.mdkib.2021@lpnu.ua (T.U.); yurii.o.sierov@lpnu.ua (Y.S.)

**Keywords:** recommender system, educational technologies, university admission campaign, academic specialty selection, decision-making system, intelligent service

## Abstract

In this article, we provide an approach to solve the problem of academic specialty selection in higher educational institutions with Ukrainian entrants as our target audience. This concern affects operations at universities or other academic institutions, the labor market, and the availability of in-demand professionals. We propose a decision-making architecture for a recommendation system to assist entrants with specialty selection as a solution. The modeled database is an integral part of the system to provide an in-depth university specialties description. We consider developing an API to consume the data and return predictions to users in our future studies. The exploratory data analysis of the 2021 university admission campaign in Ukraine confirmed our assumptions and revealed valuable insights into the specifics of specialty selection among entrants. We developed a comprehension that most entrants apply for popular but not necessarily in-demand specialties at universities. Our findings on association rules mining show that entrants are able to select alternative specialties adequately. However, it does not lead to successful admission to a desired tuition-free education form in all cases. So, we find it appropriate to deliver better decision-making on specialty selection, thus increasing the likelihood of university admission and professional development based on intelligent algorithms, user behavior analytics, and consultations with academic and career orientation experts. The results will be built into an intelligent virtual entrant’s assistant as a service.

## 1. Introduction

The problem of professional orientation is acute in Ukrainian higher schools, especially for university entrants when choosing a specialty to obtain a degree. After all, the reasons for this are clear:

- There is an inevitable information overload with information about lots of educational programs.
- The lack of a single system capable of meeting the information needs of entrants and helping to determine the most appropriate higher school.
- Complete or partial incomprehension of how the acquired knowledge will allow professional and personal development.

Globally, particularly in Ukraine, this problem is only becoming more widespread. Several related theoretical studies are merely one puzzle of the big picture that has not yet been compiled. Domestic researchers focus solely on specific aspects of this problem but do not describe it globally as a complete picture. Thus, there is still no end-to-end solution in the context of Ukrainian higher education. In addition, researchers have not agreed on a universal set of methods and technologies that will be most optimal to solve this problem, as it is individual for each state with its specifics and characteristics in science and education areas.

According to the research of Urdaneta-Ponte M., Mendez-Zorrilla A., Oleagordia-Ruiz I. (Agarwal et al. 2022), who executed a systematic literature review on recommender systems for educational purposes, 20% of all developments are concentrated in European studies. According to the study, such systems will provide relevant information to meet the target audience’s needs. Researchers analyzed four academic research databases and collected a total of 2537 papers. This article provides an understanding of the following key points and specifies the purpose of our recommender system:

1. Education type.
2. Academic aspect covered with the recommender system.
3. Target audience.
4. Methods for the recommender system development.
5. Platform to serve the users.

Correspondingly, at this stage of writing the article, our recommendation system focuses on the recommendations for traditional higher education specialties: (1) namely, the choice of university specialty; (2) our target audiences are university entrants, pupils enrolled in final school terms, and school graduates; (3) we will describe the development methods in the next section of this article (4). To provide recommendations, (5) an online platform will be delivered as a service.

Our study aims to model the end-to-end recommendation system for the target audience of Ukrainian university entrants. Additionally, we are significantly interested in a comprehensive comparison of our development with current solutions of other countries’ recommendation systems for the same educational purposes. As a result of modeling, a service model will be developed to provide valuable information for the entrants regarding the admission campaign in Ukrainian higher education institutions.

The article is divided into multiple sections. In the Introduction, we describe the problem with specialty selection globally and in Ukraine and its impact on various fields. The features of our solutions are provided in this section as well. The Literature Review section contains recent study analyses based on the Preferred Reporting Items for Systematic Reviews (PRISMA) method. We include and briefly define available solutions similar to the one we aim to develop. The Materials and Methods section is divided into two subsections. In the first one, data and user specifics, we identify critical requirements for collecting information. User input and classification are added to structure the target audience and collect insights for improving and developing our solution. The second subsection, called data processing within the recommendation system, explains how the collected information and decisions will be processed. In the Results section, we perform an exploratory data analysis of the 2021 university admission campaign in Ukraine and compared user statistics based on a set of attributes. In addition, association rules mining for university specialties selection is part of this section with a relevant collection of metrics. After analyzing the obtained outcomes, we describe the main insights and conclusions at the end of the Results section. The Discussions section contains research ideas and considerations for further developments. We include there several other fascinating findings. In the Conclusions, we briefly summarize the current state of the solution, its benefits, and demand in education and various domains.

The end goal of our system is to simplify and automate the assistance to entrants in deciding on the choice of academic specialty in universities.

In recent years, we should highlight that there has been an overkill of entrants for popular but not in-demand specialties. However, we have observed a shortage of university applicants and enrolled students for lesser-known programs in need in the labor market. Another fundamental reason and problem that needs to be solved is the balance of distribution of admitted entrants in the university specialty. The difficulty is not only to recommend an appropriate specialty but also to meet the entrant’s expectations and personal preferences.

Ultimately, according to the statistics of higher education institutions’ admission campaigns in previous years, there is a clear trend that most entrants apply to the same academic specialties for the following reasons:

- Do not know about other less popular but in-demand specialties.
- These well-known and favored ones seem pretty exciting and promising.
- Do not understand labor market trends and demand for existing academic university specialties.

In fact, due to this problem, many popular specialties are less in demand in the country and the world, as the number of graduates is excessive. While professions are in demand in the labor market, there is a shortage of students of corresponding specialties in many cases. For example, according to the statistics of the Ukrainian admission campaign in 2021, more than 1 million e-applications were submitted for a bachelor’s and master’s degree based on a complete general secondary education.

Almost 164,000 entrants submitted electronic applications through the single state electronic database on education (SSEDE). Most applications were submitted for law, managerial, humanitarian, and IT specialties. The ranking by the number of applications also includes pedagogical specialty—014 “Secondary Education”. However, we note a significant shortage of applications in science, technology, math, and some humanities academic specialties. The government has increased the number of state-funded student places for entrants to apply to these specialties, but this is not enough. For clarification: a state-funded student placed in a university means that the scholar does not pay for the tuition and is eligible to receive an academic scholarship. Simply put, the tuition for such students is covered by the government. We assume that solving this problem will provide the labor market with qualified professionals, stimulate economic growth, and help overcome unemployment.

Globally, this will solve the skill gaps problem and meet the needs of the labor market for specialists in various fields and domains. The developed recommendation system is more than labor market demand forecasting, reflecting the quantitative need for workers in a particular industry. However, the latter is not the primary deciding factor in choosing a specialty. Our solution will be a point of communication with the applicant and a virtual consultant to choose an academic specialty and provide the necessary information about it. Universities will also benefit from this. In particular, specialties with an overflow of enrolled students will reduce the load in various educational aspects. Therefore, the saved resources can be efficiently used for research projects and management optimization. At the same time, it will be possible to solve the shortage of entrants in other less popular specialties. This balance optimizes the management of curricula, higher education institutions, and processes.

The remainder of this study is represented as roadmaps. The related work in Section 2 includes Preferred Reporting Items for Systematic Reviews (PRISMA) Analysis and Recent Studies Analysis. Data and user specifics and data processing within the recommendation system is described in Section 3—Materials and Methods. Section 4, Results, presents the research results based on the association rules mining approach. Section 5 discusses the obtained results, especially the efficiency of the suggested solution. Finally, the authors conclude this work in Section 6.

## 2. Literature Review

### 2.1. Preferred Reporting Items for Systematic Reviews (PRISMA) Analysis

During the Covid pandemic, there was a dramatic transformation in the world’s vectors in academic activities. In the wake of this change, many scholars have become interested in researching these educational activity shifts. The authors examined the existing research papers according to the PRISMA method in the two largest authoritative academic research databases, Scopus and Web of Science, to thoroughly analyze the available studies.

**Figure 1:**
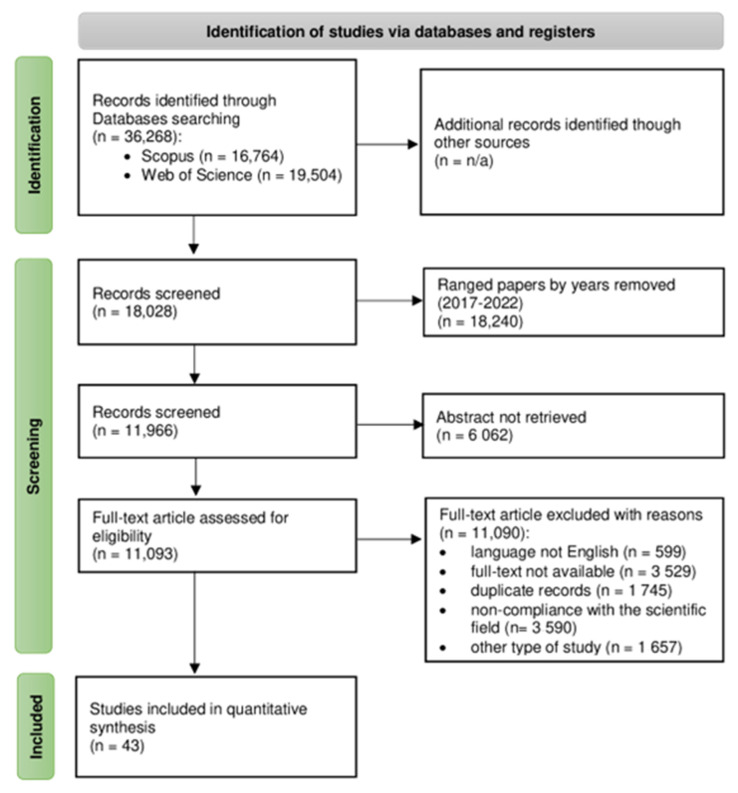
The PRISMA flow diagram for systematic reviews, which included TITLE-ABS-KEY (recommendation AND system AND education) search results of Scopus and Web of Science databases.

The analysis progress and results are represented with the PRISMA flow diagram for systematic reviews (see Figure 1). A query was created to select a set of records in Scopus and Web of Science databases: TITLE-ABS-KEY (recommendation AND system AND education).

The search results of Scopus and Web of Science databases are filtered with such parameters:

- Publication Years: 2017–2022.
- Document Types: Articles.
- Access: Open Access.
- Languages: English.

In addition, we applied additional filtering to research areas.

The following problems have been studied in selected PRISMA research papers:

- Higher education quality assurance (Alakbarov 2021; Allayarova 2019; Asare et al. 2021);
- Knowledge-based recommender system (Barabash et al. 2021; Barón et al. 2015; Bin-Noor et al. 2021; Brunello and Wruuck 2021);
- E-learning and distance learning (Bukralia et al. 2015; Burman et al. 2021; Casselman 2021; Chahal et al. 2020);
- Inter-professional education (Cheng 2017);
- Network course recommendation system (Cinquin et al. 2019; Dhar and Jodder 2020; Dolgikh 2021; Ehimwenma and Krishnamoorthy 2021);
- Modern information communication technologies in the higher education sector (El Gourari et al. 2021; Elahi et al. 2022; Ellyatt 2021; Elumalai et al. 2019);
- Transformation of education during the COVID-19 pandemic (Erridge 2006; Ezz and Elshenawy 2020; Fedushko and Ustyianovych 2020);
- Digitalization in higher education (Gao et al. 2021; Garay-Jiménez et al. 2021; Gil et al. 2019);
- Personalized recommendation system for learning resources (Habib et al. 2021; Ibrahim et al. 2019; Karan and Asgari 2021; Khan and Ramzan 2018; Khosravi et al. 2021);
- Artificial intelligence techniques for education tasks solving (Kim Rosemary et al. 2014; Levchenko et al. 2020);
- Machine learning techniques for education tasks (Korzh et al. 2014; Lee and Jung 2021; Li and Kim 2021; Mansouri et al. 2021);
- Self-learning systems development (Márquez-Vera et al. 2016; Mircea et al. 2021; Muljana and Luo 2019);
- Mobile Computing Education System (O’Neill 2018; OECD iLibrary 2022);
- Internet of Things in higher education environment (Olszewski and Siegel 2019).

### 2.2. Recent Studies Analysis

The amount of information about educational programs in universities worldwide, including Ukraine, is growing exponentially, creating information overload for entrants and pupils who intend to obtain higher education degrees. This information is not fully consolidated, which makes it impossible to adequately search for and process it in a single system for each country. Today, the primary sources of information about university academic programs are provided through the following communication channels:

1. Close contacts of the entrant (parents, friends, and various acquaintanceships);
2. University representatives, in particular professionals responsible for vocational guidance;
3. Official pages of a Higher Education Institution and its structural subdivisions in social networks;
4. Official websites of a Higher Education Institution;
5. Printed sources of information (leaflets, flyers, and magazines) about the educational institution and its programs;
6. Web forums and online blogs with ratings, descriptions of Higher Education Institutions, and academic programs;
7. Channels with educational information in online communication services (e.g., Telegram, Slack);
8. Conferences, meetings, career guidance events, and open days.

These are vital channels that provide entrants with data on educational programs at universities. However, the limitations and availability of the information received and its bias should be considered. For example, each channel may provide completely different information about the same or several specialties at various higher education institutions, including foreign ones. The entrant who received this information is not always able to qualitatively independently document, store, process, and view it in a consolidated repository.

A well-designed information system is becoming a requirement for a higher education institution. Digital information, technical leadership, enterprise architecture, and data-driven approaches are needed to successfully implement such a system (Pawade 2021). Such an approach is very relevant for our system’s development because it allows the creation of flexible and scalable architecture and adequately manages content for administrators. The factors mentioned above are helpful for our system’s development and for universities to meet informatization/digitalization challenges and build robust information processes (Prodanova et al. 2021).

Research by Song (Qassimi et al. 2021) shows the usage of wireless communication networks to provide personalized teaching resource recommendations. This approach is auspicious since data for predictions might even be streamed in (near) real time. According to the author, the application of such a method leads to lower errors and improvements in the dataset sparsity. Even though this recommendation system has shown advancements, privacy concerns might be raised because student resource usage data is collected. Correspondingly, not all users will agree to this.

Similarly, we consider tracking entrants’ university applications from open access resources but only on their agreement. The advantage of our system over the cited one is the usage of multiple models and recommendation methods. Also, we strive to understand entrants’ motivation and interests through collecting feedback and relevant input.

Cloud technologies are becoming increasingly popular, resource-efficient, and reliable. Deployment of the recommendation system (Ramírez-Montoya et al. 2021) as a service on the cloud platform contributes to information security and a high percentage of availability, depending on the chosen provider of cloud technologies. Best practices for this process are presented in the article by Dheeraj et al.

Identifying significant hidden patterns among the data of online learning system users is valuable in educational technology. Research on personalized course recommendations is significant for developing advanced e-learning systems. The article by Z. Yuwen and others presents the latest model of recommendations for learning (Samin and Azim 2019). The solution utilizes clustering and machine learning methods. Student clusters are formed based on the similarity of user features, and the model of long-term memory (LSTM) is studied to predict their possible learning directions and effectiveness. After accepting the data processing results, the most relevant educational direction is selected and recommended to the user.

Other crucial studies describe the effectiveness of experiment series with learning resource data sets. The results of the experiment show that the proposed methods are able to give valuable recommendations for appropriate areas of student learning with significantly improved knowledge outcomes in terms of accuracy and efficiency compared to previous similar studies (Sason and Kellerman 2021; Sharpe et al. 2019; Sinclair et al. 2019).

It is well-known that trends in education are constantly changing, forcing universities to deal with new challenges, and to enhance existing and develop new educational programs and paths. Recent research by Ramírez-Montoya, M.S, and others claims that artificial intelligence, a high level of organizational flexibility, and e-learning popularity are already observed or present soon as trends in the education field (Soe-aye and Rillera 2022). Thus, we should be aware that our recommendation system will become an influencing tool for entrants and future students. It is crucial to perform constant monitoring and control of recommendations made and how they affect users’ choices.

In a similar study by Ezz and Elshenawy, a multi-class binary classification machine learning technique predicts future college departments. The model aims to determine students’ performance in a specific program in the Faculty of Engineering (Song 2021). Here, the decision on a college program is made based on the predicted performance. Our research does not use this approach to recommend specialties, but we include many other crucial factors, including school performance and final exam results. However, we consider this a feature for users to predict performance on a specific specialty. Still, this must be done very carefully to avoid bias. Because students’ performance highly depends on multiple factors, making inaccurate predictions can lead to flawed conclusions by users.

The studies mentioned above focus on solutions for a particular institution, a form of study, and a country; thus, they cannot be wholly integrated into the Ukrainian higher education system. Moreover, some of the algorithms used are not suitable for working with sparse datasets as we face such problems. Nevertheless, we consider it reasonable to adopt the experience of foreign specialists, apply our custom development, and improve it to the higher education systems of Ukraine. Another vital difference between our solution and other studies is the usage of multiple decision-making algorithms and models. Depending on the previous user experience with the system, a corresponding recommendation method is used at each stage. Furthermore, we consider applying multiple recommendation models on a specific step using A/B testing to determine the most accurate one. An independent framework for university specialties classification is a subsequent distinction of our system. This framework will be created in cooperation with subject-matter experts and reflect the trends in education. However, the main advantage is determining similarities between specialties based on a set of features. Even if a new specialty is established, this framework can be applied to classify it and recommend it to the entrants.

We assume that an increased interest in the recommender systems for education and educational technologies overall is due to the following factors:

1. Transition to distance learning.
2. Rapid development of computing capabilities.
3. The need to modernize education.
4. Development and availability of cloud computing.
5. Big data opportunities for academic process optimization.

The analyzed studies significantly contribute to the research topic and, when applied appropriately, can lead to a state-of-the-art development to support the decision-making of university entrants.

## 3. Materials and Methods

### 3.1. Data and User Specifics

As of the writing of this paper, higher schools in Ukraine train specialists in 76 areas and 584 specialties. The number of areas is constantly being modified and changes with the labor market. Accordingly, prospective students have plenty of options to choose from. However, it is often difficult to predict where and for whom the entrant will work after graduation from the specialty’s name or description. According to the 2022 admission campaign rules, the number of possible applications for the state-funded form of education is 5; for commercial—up to 20. In 2021, this value was 5 and 30, respectively.

We observe a decreasing trend in the allowed number of applications submitted for university entrants. This adjustment made by the Ukrainian government leads to a more thorough and responsible choice of specialties and, correspondingly, future occupations for the entrants themselves than during previous university admission campaigns.

However, the number of specialties is quite large, while the number of possible submissions by an entrant is much lower—up to 25 altogether. This actuality confirms that we will have to deal with a sparse dataset (Stukalo and Lytvyn 2021) based on which algorithms will be trained. Supposing an entrant submits three applications only on a state-funded form, then there are only three entries in the data set, and the total number of possible entries is 584. So, theoretically, we have five hundred eighty-one blank entries for this specific entrant.

Hopefully, some algorithms, including Factorization Machines (Sulaiman et al. 2019), successfully deal with sparse dataset problems. Models based on these algorithms should be trained and tested to provide accurate recommendations to university applicants (Tavakoli et al. 2022).

Table 1 below shows our possible target audiences of entrants. Accordingly, the widest choice of specialty is for those entrants who have not yet registered for the final exams, called External Independent testing (EIT) in Ukraine. This group of entrants focuses on the specialty rather than on the EIT certificate availability required for university admission and suitable only for specific specialties.

Entrants officially register for EIT, selecting totally up to 4 school subjects to be taken on the examinations approximately 4–5 months before the exams themselves. After the subjects are selected, entrants account only for those specialties where EIT certificates with these subjects’ results are applicable. So, this study will focus on the two groups of applicants who have not selected school subjects for EIT yet. One main reason for this decision is the possibility of recommending a wide range of specialties according to the entrant’s interests instead of limiting them to the ones with applicable EIT subjects. Nevertheless, we still find it appropriate and vital to research other target groups in future studies.

In order to provide recommendations, it is necessary to collect data from applicants to study their fundamental interests and desired areas of study. Table 2 lists the common questions to be used in the system.

Proper collection and processing of this data will provide accurate recommendations and user clustering and classification.

### 3.2. Data Processing within the Recommendation System

Figure 1 shows the entity relationships chart of the university specialties database. Accordingly, the key table is “Specialties,” which contains basic information about a particular specialty of the university, the tuition, description, main field, humanitarian and technical ratios, and statistics of the previous admission campaign. One specialty can contain many hard and soft skills in its description. Skills will be selected based on their greatest relevance and uniqueness for a particular specialty.

These one-to-many relationships in the figure are implemented through the appropriate intermediate tables. Each industry has at least one sub-field with priority in the labor market, determined by relevant research and/or government agencies. Accordingly, one sub-field can be paramount in many specialties. The table with alternative specialties will contain a record ID, the primary specialty, and alternative specialty IDs. The alternative specialty is similar to the primary one. Keywords, represented with a corresponding table, will allow the entrant to focus on the main disciplines, courses, and fields of science offered for study. We use an intermediate table to connect the Specialties and Keywords tables because one specialty can contain many keywords, and one keyword might be used in many specialties.

**Figure 2:**
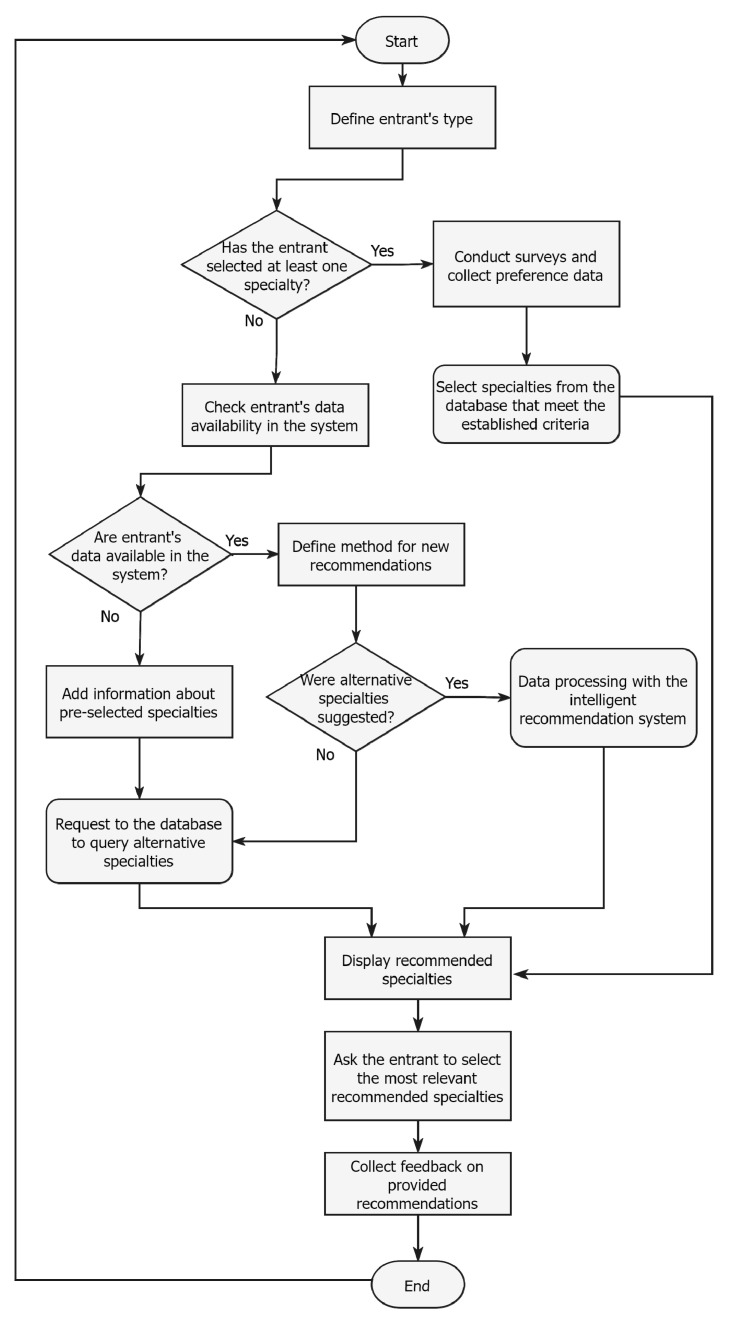
Specialty recommendation system algorithm of actions.

Figure 2 represents the algorithms of action of the recommendation service for the entrants. The workflow of how decisions will be made on the specialty selection includes machine learning and data mining methods. Data querying with further filtration is one of the initial processes to obtain the entrant’s answers and recommend an appropriate specialty. 

The first stage aims to identify the entrant’s type so that the algorithm can know to which target audience the person belongs. Afterward, whether the entrant has already selected one or more specialties should be defined. If yes, questions are provided by the system to determine skills, knowledge, and interests. When moving forward with this path, a query to the database is sent with filtering parameters to meet the entrant’s request.

**Figure 3:**
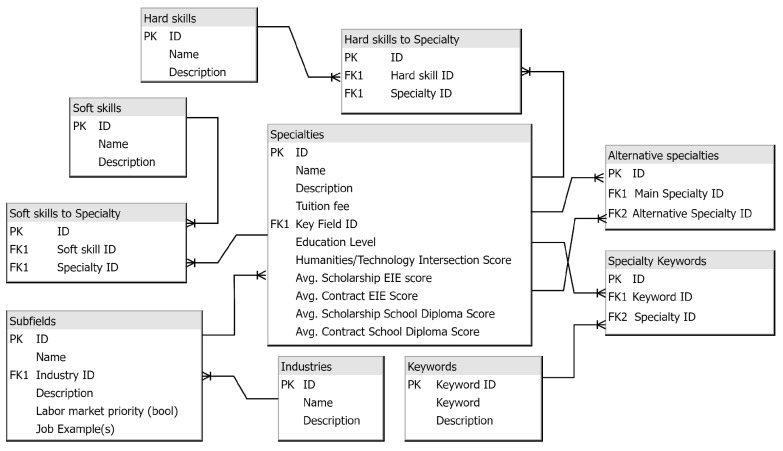
Specialties Database Entity Relationships.

Otherwise, the system checks if the entrant has used the service earlier and returns this user data. In case the system has data on the entrant’s previous specialty selections, a recommendation algorithm is defined to provide results. In this situation, only if alternative specialties were already delivered from the database, as mentioned in Figure 3, an intelligent algorithm based on neural networks performs calculations and returns recommendations. 

If not, we go with the other method and select the most appropriate alternative specialties from the table. The same method with alternative specialties will be applied to users that have not provided their choice of specialties earlier to the system once this data gap is filled.

In the pre-final stages, the system would output the recommendations and ask the user to select those that are the most interesting and appropriate to the entrant. Feedback is collected to improve the system and provide more valuable service than before.

After a particular time, the user might consider using the system again to obtain new recommendations and notifications about the experience.

Logging and monitoring system performance, user behavior, and models’ accuracy are required for further optimization and control. Therefore, a module for operational data streaming to a centralized information and event management system should be deployed at each step of the recommendation system.

## 4. Results

### 4.1. 2021 University Admission Campaign Analysis

To identify key admission campaign factors, model the probability of university admission, and develop an advanced understanding of the problem, we conducted an exploratory data analysis of the 2021 university admission campaign dataset for the Bachelor’s degree based on primary general secondary education. Data were obtained from the Unified State Electronic Database on Education (Tawafak et al. 2021). The total number of observations is 1,056,574, including 166,961 unique entrant identifiers. That is, each entrant submitted 6.32 applications on average.

The exploratory data analysis provided answers to the following questions:

1. What are the most/least popular specialties among university entrants in Ukraine?
2. According to the government, what are the percentages of applications and entrants admitted to study in specialties with special state support status, i.e., in demand?
3. Which period of the admission campaign is the most emphasized for entrants to get into university?
4. How to increase the probability of being admitted to a state-funded place?
5. Is there a statistically significant difference among entrants admitted within various priorities?

The analysis showed that 90.7% of entrants entered higher education institutions to obtain a bachelor’s degree. Among those who entered, 35.4% got admitted to the state-funded form of education, basically tuition-free education. Of the 110 specialties applied for, 58 have special state support. However, only 21.49% or 227,119 applications were submitted for such specialties. About 45,800 entrants are enrolled in such specialties—30.2% of all enrolled or 27.4% of the total entrants. We managed to find a rather exciting insight: among the entrants enrolled in the tuition-free education format, more than 53% of entrants belong to government-supported specialties. However, if we consider the contract form when students have to pay for tuition themselves, only 17.5% of entrants are admitted to such specialties. This finding indicates that an entrant is unlikely to enter a special state support specialty if a tuition fee is required.

**Figure 4:**
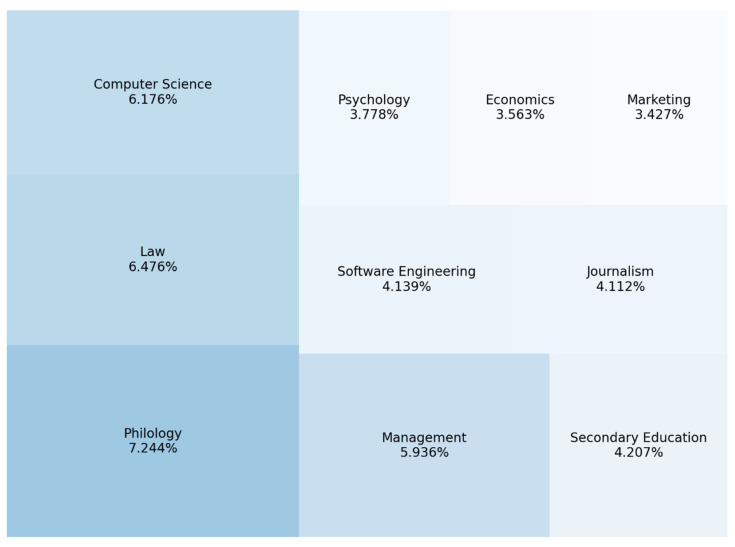
Top 10 most popular specialties among entrants during the 2021 admission campaign.

We determined the popularity of a specialty following the number of applications submitted by entrants. According to Figure 4, the apparent specialty leaders in popularity are philology, law, computer science, management, and secondary education. For example, suppose there is a shortage of software engineering or computer science specialists, secondary education, the state and global industry, and government agencies. In that case, there is a surplus of specialists in others. Among them, only one has state support—“Secondary Education”.

**Figure 5:**
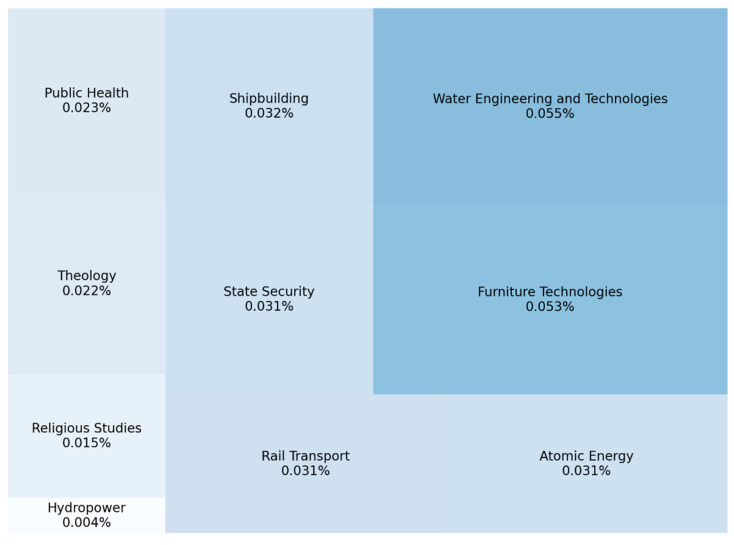
Top 10 least popular specialties among entrants during the 2021 admission campaign.

Figure 5 shows the least popular specialties among Ukrainian entrants, particularly Public Health, Rail Transportation, Atomic Energy, State Security, Hydropower, Shipbuilding, Theology, and Religious Studies, Woodworking and Furniture technologies, and Water Engineering. Seven out of ten are in demand in the state and industry and critical for technical and industrial development.

We can conclude that the problem with choosing appropriate and in-demand specialties and the problem of generally having highly qualified specialists in the future exist. This deduction again confirms the need to develop a system for providing information and recommendations to entrants. According to many recent studies, the lack of specialists in certain areas causes economic damage to states. This situation is especially critical during the COVID-19 pandemic (The Economist 2022; Tømte et al. 2019; Tungpantong et al. 2021; Unified State Electronic Database on Education 2022) and the ongoing war in Ukraine and its economic implications (Urdaneta-Ponte et al. 2021; Valkanas and Diamandis 2022).

According to the latest admission campaign in the summer of 2021, application submission to get admitted to a higher educational institution in Ukraine took place from 15 to 23 July. 

**Figure 6:**
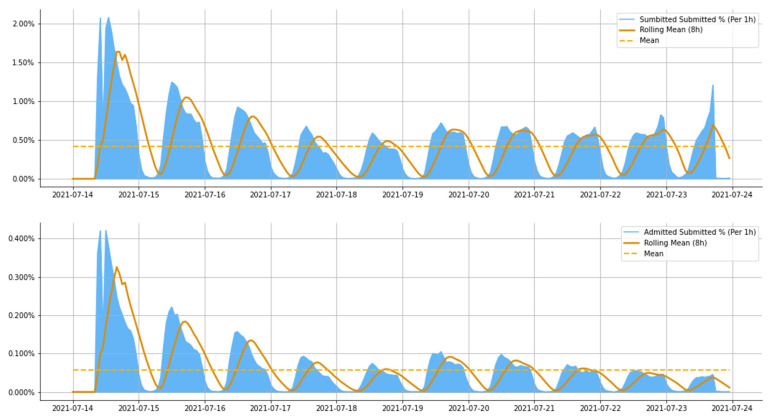
Application submitted/admitted percentage per hour during the entrance campaign.

Figure 6 clearly shows the following trend: the first three days were the most active and busy for the application submission system, and about 45% of applications were submitted. There is a gradual decline in activity, which resumes from day 6. Only about 7% of all applications were submitted on the last day. We see the percentage of enrolled entrants that submitted applications on a particular day on the same chart. We found out that on the first day, out of more than 20% of applications, only 3.25% determined an entrant’s enrollment; accordingly, the remaining 16.75% are, relatively speaking, “empty” applications for which enrollment did not take place, and the selection committee rejected them.

**Figure 7:**
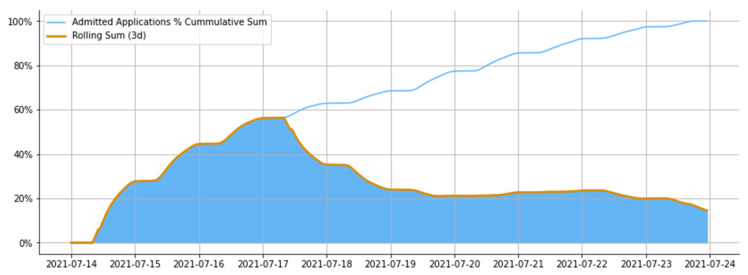
Admitted Applications by Submission date: cumulative sum.

Another insight we found is that the entrants submitted 62% of all admitted applications during the first four days of the admission campaign. This finding shows cumulative and three-day period rolling sums in Figure 7. We can infer that those entrants mainly get admitted by one of their first submitted applications.

Furthermore, as we see, the rolling sum followed the cumulative sum until the first half of 17 July 2021; afterward, a decline in the rolling sum was observed. This observation confirms our assumption that applications submitted on the first day of the entrance campaign are likely to be the most impactful and allow the entrant to get admitted to the university.

The Ukrainian rules of the admission campaign have a particular specificity: to get admitted to a state-funded place, it is necessary to indicate the application priority from 1 to 5 set by the entrant. Priority is the order of applications from 1 to 5, where 1 indicates the highest primacy and determines the order of entrant’s preference regarding the university/department. This rule applies only to state-funded forms. The priority of the applications specified by the entrant cannot be changed after applying. All other lower-priority applications are automatically canceled on the final day if a specific priority admits the entrant. For applications with priorities higher than the admitted one, it is offered to decline that one and get accepted on those with the requirement to pay tuition.

We found out that a significant number of entrants (43.5%) entered universities as a priority. “No priority” means that the entrant got admitted on a paid form of education. Thus, selecting specialties and setting priorities is critical and decisive for further professional and personal development. Further, we researched the probabilities of getting admitted to a state-funded place and the correlation of categorical data with the final competitive score.

**Figure 8:**
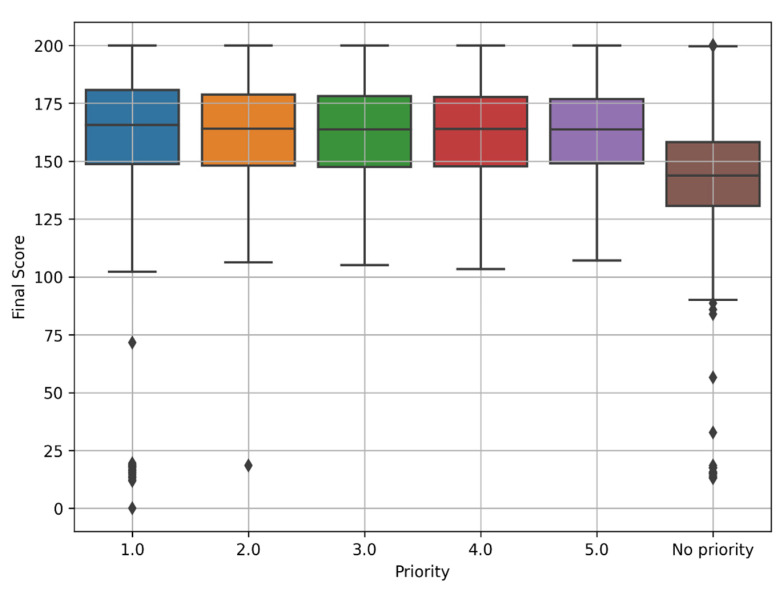
Final score box plot by admission priority.

The final competitive score is a comprehensive assessment of the entrant’s achievements, calculated based on entrance examinations and other competitive indicators with an accuracy of 0.001, following each educational institution’s general admission terms and admission rules. At the same time, we found that the final competitive score of entrants who applied for a paid form of education has lower quartiles and more outliers than those who applied for a state-funded format according to the established priority (Figure 8). Final competitive scores for priorities 1–5 are almost in the same range and indicate that the competitive score does not vary significantly by priority.

The cardinality of each priority per the number of applications is shown in Table 3 below. Here we consider all applications submitted by the entrants. We obtained another exciting finding: with the increase in the priority number, the less percentage of applications got admitted. As can be seen, most admitted applications have priority, which means that the entrant is likely to get into university on the top selected specialty. Furthermore, as we can see, only 14.23% of applications are based on which university admission occurs and determine future specialty for an entrant. Among applications with priority set to 1, 50.96% have the status “Admitted,” which is exceptionally high.

**Figure 9:**
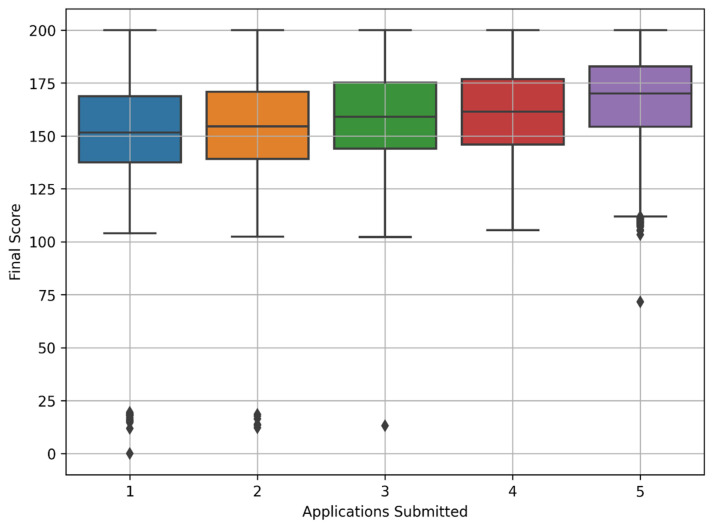
Final score box plot of admitted applicants by their applications submitted count.

In Figure 9 below, we observe the following pattern: the more applications entrants submit for a state-funded place, the higher the final competitive score they managed to achieve for university admission. This discovery is quite noteworthy and needs deeper analysis.

This study also compared entrants according to the number of applications, mainly how it affects the admission probability for a tuition-free academic place. The results presented in Table 4 contain statistics on entrants who applied to state-funded places. We note that among entrants applying for tuition-free education, 75.8% managed to get admitted to either this or a paid form. The most significant number of entrants (54.88%) submitted five applications—this is the maximum number that can be submitted for a state-funded place at the university. At the same time, this category of entrants had the highest probability of admission among this local category (96.63%) and those who applied for tuition-free forms (45.49%). The local probability of joining a particular category increases with the number of applications submitted. Let us consider the entrants with the number of applications between two and four. We see that the probability of admission also increases among all entrants and in their category.

Accordingly, the last column of the table, “Admitted% out of the total,” shows what percentage of entrants who applied for the state-funded education form still got admitted. This obviously answers the question, “How to increase the probability of getting admitted to a state-funded place?”:

- Submit as many applications as possible to the tuition-free form.
- Set priorities correctly.
- Get the highest possible final competitive score.

Adherence to these points will increase the likelihood of the above question fulfillment. The developed recommendation system will allow entrants to effectively choose a specialty, set priorities, as well as motivate them and set the necessary goals for successful university admission.

### 4.2. Association Rules Mining

For the system to provide high-quality recommendations, it is necessary to understand entrants’ specialty itemset choices. This knowledge will address the following issues:

1. What specialty Xj to recommend to entrants who applied for Xi?
2. What alternative specialties can an entrant apply for, given his current choice? How to display it in a data set?
3. What is the relationship between the admission probability and specialty itemsets?

The usage of association rules mining allowed us to answer these questions. Applying this and other data mining methods in education remains relevant and has enough use-cases (Villegas-Ch et al. 2021; Walsh et al. 2022).

Data were transformed in a Python virtual environment using a Jupyter Notebook to conduct association rules mining. A data set with parameters such as the entrant’s ID and the specialty’s name was read. These two columns were transformed into an information table, where the column names are the specialty names, and each row represents unique entrant IDs. Accordingly, if the entrant has applied for a particular specialty, the corresponding cell contains the value 1; otherwise, 0.

We used the mlxtend python library, namely apriori and association_rules methods from the frequent_patterns submodule. We applied the apriori method with such parameters: ‘min support’ equal to 0.007 gave us a transformed data set as outputs with 348 specialty itemsets. Our goal was to get as many itemsets as possible; the low support threshold was set. Based on this dataset, we used the association_rules method with minimal confidence thresholds set to 0.03. As a result, we obtained a consolidated data frame with the following metrics for each itemset: antecedents support, consequents support, itemset support, confidence, lift, leverage, and conviction. The total number of generated association rules was 1115.

We determined the most valuable samples based on numerical metrics and itemset uniqueness. Market needs have been taken into account. The results are represented in Table 5.

**Figure 10:**
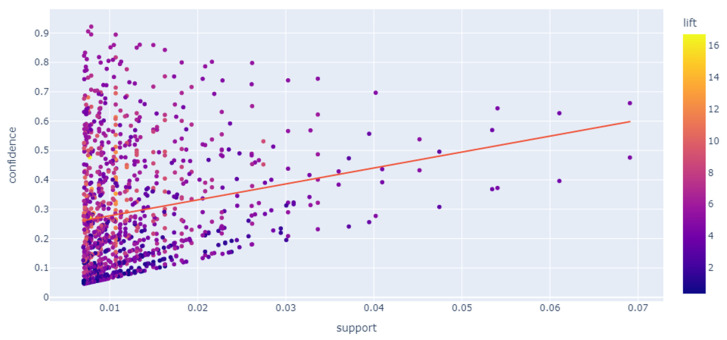
Generated association rules metrics correlation.

A scatter plot is generated in Figure 10, which shows the correlation among each association pair’s support, confidence, and lift. As we can see, there is a slight correlation between confidence and support. As support increases, confidence increases as well. However, it should be noted that many rules may be in the same low support range, but the confidence value varies. The reason is the distribution of the observations for each specialty in the data set: less popular specialties tend to have low support, but their co-occurrence with another specialty is quite likely, contributing to high confidence. The R-squared score is 0.041.

An example is the antecedent “System Analysis; Software Engineering” and consequent “Computer Science,” where support and confidence are 0.013416 and 0.860215. This itemset contains three entities in total—accordingly, the more elements, the lower the support. The lift indicator, which forms a measure of the rule importance, tends to increase with increasing confidence and decreasing support.

**Figure 11:**
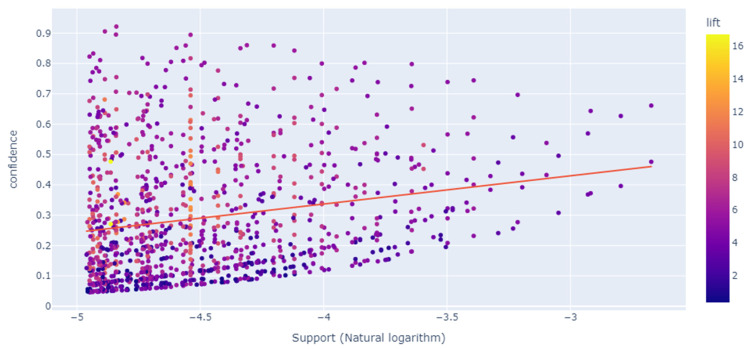
Generated association rules metrics correlation with a logarithmic support value on the *x*-axis.

We applied the natural logarithm to reduce a wide range of the support value to a manageable size. The results are depicted in Figure 11.

Consequently, we can see a slightly better dispersion of the support values and correlation with confidence. However, the R-squared score was slightly reduced to 0.039 compared to the previous value. We ensured that most of the points with low confidence have low support. These are the unpopular specialties overall and in their itemsets. Those with high confidence and low support tend to be not very popular in general.

Nevertheless, within the itemsets, their popularity might be higher. Their lift is also comparatively high, which means such rules are valuable. Itemsets with high confidence and support up to a certain threshold, which in our case is −4 on the *x*-axis, tend to have a relatively high lift. A minor increase in support occurs along with confidence. In addition, we observed outliers with high support and confidence. These are the popular specialties likely to occur both overall and within itemsets.

We consider two main applications for the association rules mining technique to be integrated into the recommendation system:

- generate reports on specialties selection among entrants for internal use to gain insights about university admission campaigns, share them with the development team and all other subjects interested in this topic (academic departments, faculties, and universities) at their request.
- Build a model for specialty selection based on this technique. Its validation and comparison with other furtherly developed models might be performed using methods such as A/B Testing.

After analyzing the association rules, we can draw the following conclusions:

1. Entrants who choose popular specialties tend to choose the same popular alternatives.
2. In 98% of cases, entrants choose alternative specialties from the same field; we did not find an itemset with humanitarian and natural or technical specialties.
3. We found several associative rules that contain technical and managerial specialties; this reflects the market need for managers with technical backgrounds or technology workers with advanced management skills.
4. The choice of alternative specialties among entrants is exceptionally high-quality but needs improvement.
5. Many entrants do not understand the difference between similar specialties in one field; correspondingly, they tend to apply for as many as possible with the exact keywords in these specialty names.

## 5. Discussion

The results of this and other analyzed studies confirm that the education field is specific not only in each country and region but also in individual higher education institutions. One academic specialty may be completely different in another university or geographical region. Accordingly, the recommendation system in the context of Ukrainian universities and the admission campaign is critical for developing domestic and global educational technologies. We consider it appropriate to use other research on the best practices for collecting, processing, and analyzing educational data. These methods would be especially appropriate during Big Data development and integration to improve university operations.

According to the exploratory data analysis, we can say that the university’s admission campaign (Wang 2022) successfully complies with the Pareto rule (Xu et al. 2021): about 80% of applicants apply for and get admitted to about 20% of the most popular specialties. Similar findings in education with this rule are represented in several recent studies (Yahya and Osman 2019; Zhong and Ding 2022; Y. Zhou et al. 2018; J. Zhou et al. 2022).

In compliance with the research results, entrants are able to select alternative specialties to those already selected successfully. However, when it comes to primary specialty—preference is given to the most popular and advertised, which negatively affects the labor market and puts an extra burden on the corresponding departments of educational institutions. To solve this problem, we propose to develop a service for interactive selection of educational specialties and entrants’ information support. The specialty recommendation action algorithm and ER diagram are a crucial part of the specialty recommendation decision-making architecture. This service will allow an entrant to choose and describe a specialty to study at the university and interpret for the entrant how the decision is made and why the recommended specialty meets the entrant’s interests and requirements. Consequently, this solution will provide an understanding of specialty characteristics and draw more attention to in-demand but less popular specialties.

Despite the discovery that entrants manage to choose alternative specialties, we consider it appropriate to pay attention to this issue. We point out the need to form a data set that contains alternative specialties to each available one. Consultations with industry experts might be helpful to us to create such a dataset effectively. Moreover, we consider the possibility of data augmentation in order to increase the balance of relevantly selected alternative specialties and their recommendations to entrants.

Another issue we need to address is providing accurate recommendations. This issue applies primarily to popular specialties; the entrant applies for these specialties because:

1. The entrant purposefully seeks to acquire a specific profession.
2. This specific industry is considered popular, and an entrant no longer sees alternatives.

None of these options are wrong, but the latter requires less self-awareness for an entrant. Accordingly, it is advisable to solve this problem and provide more awareness in the process of choosing a specialty. The developed information service should contain an option to understand the entrant’s motivation to enroll as a student in a particular university specialty.

Analyzed research developments provide recommendations to improve the learning experience (Zhu 2021). This feature should be used in our development. Thus, the entrant receives a recommendation on the specialty choice and the opportunity to choose a key specialty itemset and receive personalized advice on succeeding in the learning pathway. This feature will further contribute to successful learning and professional development. However, implementing this functionality requires additional data collection and the development of an algorithm that can be reproduced from other scientific papers on this topic.

The feedback collection system will open up even more opportunities to improve the recommendation system. Comprehending whether the entrant is satisfied with the service and recommendations opens horizons for using various technology types, including reinforcement learning. User evaluation will be a pointer to continuous system improvement.

## 6. Conclusions

This development will emphasize the high level of educational technologies in Ukraine and the world. Not only entrants will benefit from it, but also providers of educational services (universities, online educational platforms, and academies). In particular, the latter will increase the number of applicants for degrees, users, and students, analyze the target audience, provide a more personalized learning path in the future, and create competitive educational services.

Given the results obtained, consultations for higher educational institution representatives in Ukraine, particularly departments, research institutions, and participants in career guidance campaigns, will be beneficial. These sessions will contribute to a better understanding of the decision-making process of Ukrainian university entrants and allow better targeting of individual school graduate cohorts.

For entrants, the benefits are pretty obvious: the ability to choose an educational program for themselves, considering their interests, skills, budget, and location. Additionally, one of the key goals is to provide self-awareness in a specialty to study. After all, as seen in popular specialties, many entrants apply there only because they are well-known. This pattern can have a detrimental effect on the market professionals’ quality, labor market trends, as well as entrants and their financial resources.

In further research, we suggest accomplishing the following:

1. Develop automated solutions to find similar and alternative specialties.
2. Carry out data augmentation of a successfully selected specialties itemset to provide better and more unique, personalized recommendations to entrants.
3. Identify and increase entrants’ awareness when choosing a place to study.

This problem is especially relevant during the COVID-19 crisis when, due to a lack or poor quality of communication with the surroundings, entrants are unable to attend career guidance events, get acquainted in detail, and discuss the admission rules for a particular specialty.

It is necessary to mention the military and geopolitical situation in Ukraine because the appropriate choice of specialty will support and accelerate the development and reconstruction of the country in the current and postwar periods. Given the current war situation in Ukraine and the Russian aggression, much damage has been inflicted on domestic industries, and companies must stop operations in hostile zones. In addition, many infrastructures were destroyed. Accordingly, the need mentioned above for young professionals to rebuild the country is critical. Thus, the recommendation system will allow entrants to choose those specialties that will allow them to be most helpful to the state and meet their interests and preferences. After all, a person needs to do what brings joy and, at the same time, value.

## Figures and Tables

**Table 1:** Entrants’ target audience for the recommendation problem.

Is Registered to EIT?	Is EIT Been Passed?	At Least One Specialty Is Selected?
No	No	No
No	No	Yes
Yes	No	No
Yes	No	Yes
Yes	Yes	No
Yes	Yes	Yes

**Table 2:** Entrants respond to dataset structure for providing university specialty recommendations.

Question	Expected Value	Application
Desired study fields	Textual proposed definition of industry areas from the list	Identify the key interest fields to the entrant
Desired study subfields	One or more suggested text values from the list of subfields	Identify key interest subfields to the entrant
The main goal when choosing the specialty	Career opportunities; self-development; interesting academic process; opportunity to engage in a certain type of academic activity; formal need to obtain a degree	Understanding the entrant’s motivation further to improve the service and the appropriate selection of specialties
Expectations from the educational process at the university/department	Text data from the entrant. Optional field	Natural language processing (NLP) usage to find the most similar specialties according to the similarity score between their description, keywords, and the entrant’s expectations
Technician/Humanitarian preference ratio	A numeric value indicating the entrant’s preferable specialty focus	The selection of specialties depends on their ratio of humanitarian and technical focuses. Also, we can determine whether an entrant is interested in technical, humanitarian specialties, or an intersection of both.
Already selected specialties	Specialties the entrant has selected from the dropdown	Find alternative specialties and understand entrants’ motivation and interests.
Study format	Online/full-time/part-time	Selection of specialties according to the selected study format
Priority on state-funded education	Boolean value (True/False)	Selection and sorting of recommended specialties in descending order of admission probability to study on a state-funded form
Estimated budget for tuition per year/total tuition	A numerical value representing acceptable tuition for an entrant per specified period (term/year/multiple years)	Defining specialties that satisfy the entrant’s financial ability
Minimum/average/maximum scores in the current/latest educational institution (e.g., secondary school) on a national scale	Separate numerical values. For minimum and maximum scores would be good to provide subject names	Select the most relevant specialties following the success of education in primary school. It will also help determine how a specialty complexity level corresponds to the entrant’s knowledge level
Evaluation of the provided recommendations’ relevance	Relevant/Irrelevant OR a numerical value in a specified range	Define user satisfaction for the recommender system

**Table 3:** Statistics on entrants who applied for state-funded form.

Priority	Total Applications	Total Applications %	Admitted Applications	Admitted % Out of Local Category	Admitted % Out of the Total
1	128,442	12.15%	65,455	50.96%	6.19%
2	115,382	10.92%	18,211	15.78%	1.72%
3	105,005	9.93%	9263	8.82%	0.87%
4	92,550	8.75%	6157	6.65%	0.58%
5	79,813	7.55%	4654	5.83%	0.44%
No priority	535,382	50.67	46,627	8.70%	4.41%
**Total**	**1,056,574**	**100%**	**150,367**	**-**	**14.23%**

**Table 4:** Statistics on entrants who applied for state-funded form.

Application(s) Submitted	Total Entrants	Total Entrants %	Admitted Entrants	Admitted % Out of a Local Category	Admitted % Out of the Total
1	18,562	13.69%	11,561	62.28%	8.53%
2	13,144	9.69%	8172	62.17%	6.03%
3	13,721	10.12%	9546	69.57%	7.04%
4	15,756	11.62%	11,897	75.51%	8.77%
5	74,418	54.88%	61,684	82.89%	45.49%
**Total**	**135,601**	**100%**	**102,860**	**-**	**75.86%**

**Table 5:** Association rules of university admission campaign specialties.

№	Antecedents	Consequents	Itemset Support	Confidence	Lift
1	Economics	Management	0.047	0.495	3.216
2	Computer Science	Software Engineering	0.069	0.476	4.557
3	Computer Engineering	Cybersecurity	0.035	0.428	4.570
4	Philology	Secondary Education	0.029	0.234	1.749
5	International Relationships	Philology	0.028	0.513	4.089
6	Hotel-restaurant Business	Tourism	0.027	0.531	8.760
7	Journalism	Law	0.025	0.260	2.102
8	Automation and computer-integration technologies	Computer Science	0.024	0.490	3.378
9	Accounting and Taxation	Economics	0.019	0.420	4.390
10	International Law	Law	0.017	0.614	4.958
11	Applied Mathematics	Computer Science	0.013	0.600	4.138
12	Cybersecurity	Management	0.013	0.144	0.934
12	Management	Cybersecurity	0.013	0.087	0.934
13	System Analysis	Computer Science; Software Engineering	0.013	0.425	6.154
14	History and Archeology	Political Science	0.011	0.291	7.076
15	Industrial Engineering	Electric power, electrical engineering and electromechanics	0.009	0.324	8.617
16	Culturology	Journalism	0.009	0.491	4.976
17	Biology	Ecology	0.008	0.301	7.939
18	Finance, banking, and insurance	Cybersecurity	0.008	0.106	1.138
19	Psychology	Computer Science	0.007	0.074	0.516
20	Applied Mechanics	Industrial Engineering	0.007	0.369	12.892

## Data Availability

Not applicable.
